# Silencing of peroxiredoxin 2 suppresses proliferation and Wnt/β-catenin pathway, and induces senescence in hepatocellular carcinoma

**DOI:** 10.32604/or.2023.030768

**Published:** 2023-11-15

**Authors:** XUEGANG YANG, XIANHONG XIANG, GUOHUI XU, SHI ZHOU, TIANZHI AN, ZHI HUANG

**Affiliations:** 1 Department of Interventional Radiology, Sichuan Cancer Hospital and Institute, Sichuan Cancer Center, School of Medicine, University of Electronic Science and Technology of China, Radiation Oncology Key Laboratory of Sichuan Province, Chengdu, 610041, China; 2 Department of Interventional Radiology, The First Affiliated Hospital of Sun Yat-sen University, Guangzhou, 510080, China; 3 Department of Interventional Radiology, Affiliated Hospital of Guizhou Medical University, Guiyang, 550004, China; 4 School of Basic Medical Science, Guizhou Medical University, Guiyang, 550002, China

**Keywords:** Peroxiredoxin 2, Hepatocellular carcinoma, Wnt/β-catenin pathway, Senescence, Proliferation

## Abstract

Hepatocellular carcinoma (HCC), a common malignancy worldwide, still lacks effective clinical treatment. The study aimed to investigate the oncogenes that affect the progression of HCC and their possible mechanisms. In our study, we initially confirmed a higher level of PRDX2 in the bile of HCC patients compared to those with choledocholithiasis by 2-DE, LC-MS, and ELISA. Subsequently, we demonstrated the high expression of peroxiredoxin 2 (PRDX2) in HCC based on the TCGA database and clinical sample analysis. Furthermore, PRDX2 overexpression enhanced the viability of HCC cells. And PRDX2 silencing induced senescence of HCC cells. *In vivo*, knockdown of PRDX2 significantly reduced the weight of xenograft tumors. PRDX2 also was found to activate the Wnt/β-catenin pathway by inducing β-catenin nuclear translocation. Consequently, we proved that silencing PRDX2 could inhibit proliferation and Wnt/β-catenin pathway while promoting senescence in HCC cells.

## Introduction

Hepatocellular carcinoma (HCC) is currently the sixth most common malignant cancer worldwide [1,2]. The majority of HCC cases (>80%) occur in sub-Saharan Africa and Eastern Asia [3]. China, in particular, is considered a high-risk regions for HCC, primarily due to factors such as chronic hepatitis B virus (HBV) infection or aflatoxin exposure [4,5]. Despite various treatment techniques available for HCC patients in clinical practice, long-term survival outcomes remain unsatisfactory [6]. The challenges in HCC treatment largely stem from our limited understanding of the molecular-level tumorigenic mechanism of HCC. In addition, early diagnosis of HCC remains challenging due to the high incidence of asymptomatic disease, resulting in a reported 5-year survival rate of approximately 10% [7]. Therefore, further exploration of the pathogenesis and mechanism underlying HCC is of great significance to reduce HCC-related mortality.

Recent studies have demonstrated the utility of proteomic analysis for quantifying proteins in bile and exploring potential biomarkers [8,9]. Through screening with two-dimensional electrophoresis (2-DE), we identified peroxiredoxin 2 (PRDX2) as a potential therapeutic marker for HCC. PRDX2, a key member of the PRDX family, functions as a vital intracellular antioxidant [10]. It actively participates in the cellular antioxidant defense system, effectively mitigating cellular damage caused by oxidative stress and promoting tumor cell proliferation and survival [11]. Furthermore, PRDX2 plays a crucial role in signal transduction, cell differentiation, apoptosis, metastasis, radiotherapy resistance, and other processes [12,13]. High expression of PRDX2 has also been reported in various cancer cells and tissues, contributing to cancer progression [14,15]. Increasing evidence suggests that PRDX2 has a regulatory role in tumorigenesis [14,16,17]. Several studies have also proved the involvement of PRDX2 in HCC progression [18–22], making it a valuable candidate for assessing therapeutic efficacy and cancer prognosis. However, the precise effects of PRDX2 on HCC cell senescence and the underlying mechanisms remain incompletely understood.

Accumulating evidence suggests that dysregulation of Wnt/β-catenin pathway plays a crucial role in the tumorigenesis and development of certain solid tumors and hematological malignancies [23–25]. Activation of Wnt/β-catenin pathway occurs through the accumulation of β-catenin protein in the nucleus [26,27]. It has been reported that approximately 40%–70% of HCC patients exhibit nuclear accumulation of β-catenin protein [28,29]. The activation of Wnt/β-catenin pathway results in extensive transcription of downstream target genes [30]. Moreover, research has also manifested that PRDX2 could promote the progression of esophageal squamous cell carcinoma through the Wnt/β-catenin pathway [31]. However, whether PRDX2 regulates Wnt/β-catenin pathway in HCC cells is unclear.

In this study, we conducted further investigations to explore the effects of PRDX2 on the proliferation and senescence of HCC cells both *in vitro* and *in vivo*. Additionally, we sought to verify the effects of PRDX2 on Wnt/β-catenin pathway. Consequently, our findings support the proposition that that PRDX2 may serve as a potential biomarker for HCC.

## Materials and Methods

### Patients’ characteristics

In this study, bile fluid samples were collected from 26 patients at the Affiliated Hospital of Guizhou Medical University, China, between January 2011 and August 2013. All patients were followed up for a period of 3 to 6 months, with a minimum follow-up duration of one year. Additionally, serum samples from 35 patients constituted an independent “validation” cohort. HCC was diagnosed based on the criteria established by the American Association for the Study of Liver Diseases (AASLD) [32]. None of the HCC patients received chemotherapy. Choledocholithiasis was diagnosed through magnetic resonance imaging, endoscopic ultrasound, endoscopic retrograde cholangiography (ERCP), and other diagnosis methods. This study was approved by the local ethics committee of Guizhou Medical University. The written informed consent was obtained from all participants.

### Sample collection

Bile fluid samples were obtained from patients undergoing ERCP for choledocholithiasis (n = 14) or percutaneous transhepatic cholangial drainage (PTCD) for HCC (n = 12). Up to 20 ml of bile fluid samples were collected in prechilled, ice-cold tubes and immediately centrifuged at 16000 g for 15 min at 4°C to remove cell debris, nucleic acid and mucins. The resulting supernatant was then stored in 1 ml aliquots at −80°C until further analysis. Serum samples were also collected and stored accordingly. For immunohistochemistry analysis, a total of 9 pairs of HCC and para-carcinoma tissues were obtained from the Department of Surgery, Affiliated Hospital of Guiyang Medical College, China.

### Pretreatment of bile prior to proteomic analysis

To prepare the bile samples for proteomic analysis, 1 ml of preliminary separated bile samples were chilled on the ice and mixed with 250 μl of cleanascite™ (Biotech Support Group, NJ, USA). After 1 h of rotation, samples were centrifuged to remove lipid micelles. Subsequently, the resulting supernatant was transferred to a Millipore YM-3 Centurion Filter Unit (Bedford, MA, USA), where it underwent centrifugation and filtration [33]. Finally, 200 μl of MilliQ water was added, and the centrifugation process was repeated three times, following previously described protocols [34].

### Two-dimensional electrophoresis (2-DE)

Based on previous studies [35,36], a total of eight patients (choledocholithiasis, n = 4; HCC, n = 4) were included in this study. Bile samples were mixed with pre-cooled acetone at a four-fold volume of precipitation. After incubating the mixture at 20°C for 2 h, the precipitated proteins were collected by centrifugation. Acetone was discarded, and the protein pellet was subjected to ice drying. Subsequently, protein pellets were re-solubilized using a lysis buffer containing 4% CHAPS, 0.001% bromophenol blue, 2 M thiourea, 65 mM dithiothreitol, 7 M urea, 0.5% pH 3–10 and IPG buffer. Protein concentrations were determined using the Bio-Rad DC system. Each 1 mg of purified sample was diluted to 350 μl with the buffer. Protein samples were then loaded onto Immobilized pH gradient (IPG) strips (17 cm; Bio-Rad, USA) for isoelectric focusing (IEF) with a commercial device (Protean IEF Cell, Bio-Rad, USA). The focusing step was covered with mineral oil (Bio-Rad, USA). Isoelectric focusing was performed following the manufacturer’s instructions. After isoelectric focusing, IPG strips were equilibrated by immersing them in an equilibration buffer for 15 min. SDS-PAGE separation was then performed with a vertical electrophoresis system (Bio-Rad, Protein II, USA) at a current of 5 mA/gel for 30 min, followed by 25 mA/gel for 5 h.

### Staining and image analysis

In accordance with the study [37], the gels were fixed with a solution containing 40% methanol and 10% acetic acid for 1 h. Following fixation, the gels were stained with 0.025% Komas Blue R-350 (Imperial Chemical Industries, London, UK) and subsequently heated to 80°C–90°C. For gel destaining, a 10% acetic acid solution was employed. Subsequently, the gels were scanned using a commercial imaging program (Bio-Rad Labroratories, GS-800, USA). The acquired images were analyzed with PDQuest 2D software (Bio-Rad, USA). Protein spots exhibiting a fold-change in intensity of at least 2-fold were selected, subjected to digestion, and analyzed using MALDI-TOF-MS.

### Identification of protein by MALDI-TOF/TOF-MS analysis

Protein identification was conducted following previously reported protocols [38]. Protein spots on the 2D gels were manually selected, excised, and subjected to digestion. To decontaminated the gel spots, a solution of 25 mM ammonium bicarbonate and 50% acetonitrile (ACN) was employed at 37°C, followed by ACN dehydration. Subsequently, the gel blocks were digested and treated with 25 mm ammonium bicarbonate for 8 h at 37°C. After elution, the peptides were solubilized and analyzed using MALDI-TOF/TOF-MS. The MALDI-TOF and MALDI-TOF/TOF spectra were acquired using an Ultraflex III TOF/TOF mass spectrometer (Bruker Daltonics, Bremen, Germany). Peptide mass fingerprint (PMF) data were compared with Swiss-Prot or NCBInr databases using MASCOT [26,27], and the confidence levels of the identified proteins were determined. A MASCOT scores greater than 62 indicates statistical significance (*p* < 0.05).

### RT-qPCR assay

The tissues were ground, and cells from each group were collected. Total RNA was extracted using the RNeasy Mini Kit (QIAGEN, Hilden, Germany). Subsequently, cDNA synthesis was performed using a reverse transcription kit (Promega). RT-qPCR was conducted using the SYBR-Green PCR kit (TransGen Biotech, Inc., Beijing, China). The data was analyzed using the 2^−ΔΔCt^ method to determine relative gene expression levels.

### Western blot analysis

A total of 20 μg of proteins from each patient’s bile (choledocholithiasis, n = 14; HCC, n = 12) were separated by SDS-gel electrophoresis and transferred to PVDF membranes (Millipore, USA). The membranes were then incubated overnight at 4°C with primary antibodies, including anti-PRDX2 (1:1000, Abcam, ab109367), anti-CyclinD1 (1:1000, Abcam, ab16663), anti-cMYC (1:1000, Abcam, ab32072), anti-c-Jun (1:1000, Abcam, ab40766), anti-fra1 (1:1000, Abcam, ab252421), anti-β-catenin (1:1000, Abcam, ab32572), anti-Lamin B1 (1:1000, Abcam, ab16048), anti-GAPDH (1:2000, Abcam, ab76523) and anti-β-actin (1:1000, Abcam, ab8227). Following primary antibody incubation, the membranes were washed and incubated with peroxidase-conjugated secondary antibody (1:2000, Abcam, Cambridge, UK). Subsequently, substrate development was performed using ECL chemiluminescence (Millipore, Bedford, Mass, USA).

### Immunohistochemistry (IHC) assay

HCC and para-tumor tissues were fixed, dehydrated, cleared, immersed in wax, embedded, and sectioned. Paraffin sections were then subjected to gradient ethanol dehydration and antigen retrieval using sodium citrate. After blocking, the sections were incubated overnight at 4°C with primary antibodies against PRDX2 (Abcam, ab59539, 1:50). Then, HRP-labeled secondary antibodies (1:200, Abcam) were applied and incubated at 37°C for 30 min. The sections were subsequently stained with diamino-benzidine (DAB, Maxim Biotech, Fuzhou, China) and hematoxylin (Solarbio, China; cat. no. H8070), followed by differentiation, hydration, and making them transparent. Finally, the slides were sealed, and the results were observed under a microscope (Nikon, Tokyo, Japan).

### Enzyme-linked immunosorbent assay (ELISA) assay

ELISA was performed to determine the levels of PRDX2 using a kit supplied by CUSABIO (Wuhan, Hubei, China) according to the manufacturers’ protocols.

### Cell culture and treatment

The SNU-387, PLC.5, SK-Hep-1, Huh7, and HepG2 (Purchased from ATCC) cells were cultured in DMEM (Gibco, cat. no. 1859228) supplemented with 10% fetal bovine serum (FBS, Gibco), 100 units/ml penicillin G, and 100 mg/ml streptomycin. The cells were incubated at 37°C in a cell culture incubator with 5% CO_2_. Prior to the experiment, the cells were collected for further analysis.

### Transfection

The day before transfection, cells were plated in 6-well plate with normal growth medium without antibiotics at a density of 2 × 10^5^ cells/well. On the day of transfection, when the cell density reaches 80%, the medium was replaced with Opti-MEM (Invitrogen). The DNA or Lipofectamine 2000 was mixed separately with Opti-MEM and incubated for 5 min. Subsequently, the diluted DNA and diluted Lipofectamine 2000 (Invitrogen) were gently mixed together and left at room temperature for 20 min before adding to cells.

### CCK-8

HuH-7 cells were transfected with indicated plasmids. Following transfection, cells were subjected to puromycin selection at a concentration of 1 μg/mL for 4 days before conducting the CCK-8 assay. For the CCK-8 assay, 100 μl of transfected HuH-7 cells (2 × 10^3^ cells) were transferred to each well of a 96-well plate and incubated for 24 h. Afterward, 10 μl of CCK-8 solution (Beyotime, China) was added to each well, and the plate was incubated at 37°C for 1.5 h. The absorbance values were then measured using a microplate reader.

### Colony forming assay

HuH-7 cells were transfected with indicated plasmids. After transfection, puromycin selection was applied at a concentration of 1 μg/mL for 4 days before performing colony forming assay. For the assay, 500 transfected cells were plated into 6-well plate containing medium with 10% FBS and 1% Penicillin-Streptomycin. Cells were then cultured for approximately 2 weeks, fixed with cold methanol, and stained with 0.1% crystal violet.

### Immunofluorescence

Coverslips were plated in 6-well plates, and HepG2 cells were added onto them. The following day, cells were fixed with 4% paraformaldehyde, then permeabilized by treating them with PBS containing 0.1% Triton X-100 for 15 min. The slides were washed three times with PBS, then blocked with 2% BSA for 30–45 min at room temperature, The slides were incubated overnight with the following primary antibodies: anti-PRDX2 rabbit monoclonal antibody (1:250, ab109367, Abcam) and anti-β-catenin mouse monoclonal antibody (1:200, ab19381, Abcam) at 4 degrees. The next day, the slides were washed three times with PBST for 5 min each time. Then, the slides were incubated with fluorescent secondary antibody: Goat anti-rabbit-DyLight® 488 (1:1000, ab96899, Abcam) and Goat anti-mouse-DyLight® 647 (1:1000, ab150115, Abcam) for one hour at room temperature in the dark. Afterwards, the slides were washed three times with PBST and observed using a fluorescent microscope.

### Top-flash assay

Top-flash reporter plasmid and PRDX2 expression plasmid were co-transfected into HEK293 cells, following the transfection procedure. After 2 days of transfection, a dual luciferase assay kit (Promega) was used to measure the luciferase activity of the cells.

### Experimental model animals

A total of 16 BALB/c nude mice (8 weeks old) were purchased from Jiangsu Jicui Yaokang Biotechnology Co., Ltd. (Nanjing, China). All mice were individually housed in an SPF animal laboratory. Following a period of 1 to 2 weeks of adaptive feeding, HepG2-shGFP (shGFP) and HepG2-PRDX2-shRNA#2 (shRNA#2) cells (2 × 10^6^) were separately suspended in Matrigel. Subsequently, these cells were subcutaneously implanted into the flanks of 8-week-old BALB/c nude mice. Tumor volume was monitored on days 5, 8, 11, 14, 17, and 20. This study was approved by the Research Ethics Committee of the Guizhou Medical University.

### β-Gal activity detection

HepG2 cells were treated with shGFP or sh#2 for 48 h and collected. After centrifugation, the β-Gal activity was analyzed using the β-Gal assay kit (Solarbio, Beijing, China) following the instructions provided with the Kit.

### CO-IP assay

After washing twice with pre-cooled PBS, the cells were lysed with pre-cooled RIPA buffer. The lysates were then centrifuged at 14000 g for 15 min at 4°C, and the resulting supernatant was transferred to a new centrifuge tube. The supernatant was incubated overnight at 4°C with 1 μg of primary antibody/20 μl of protein A/G beads (Abcam). The next day, samples were centrifuged at 14000 g for 15 min at 4°C. The supernatant was discarded, and the beads were washed with loading buffer. Subsequently, the beads were boiled at 100°C for 5 min, and Western blotting was performed.

### Statistical analysis

The data of this study were obtained from three replicate experiments, and statistical analysis was performed using Graph Pad Prism. The parametric tests used in this study included Mann-Whitney U-test and Student’s *t* test. Measurement data were presented as Mean ± SD. Unpaired *t* test and Bonferroni *post hoc* test were used to compare two groups of data with normal distribution and homogeneity of variance. Pearson correlation analysis was used to analyze the relationship between variables. Multiple regression analyses were performed using SPSS version 16.0 (SPSS, Chicago, IL, USA). Immunohistochemical markers were evaluated using Fisher’s exact test. Statistical significance was defined as *p* < 0.05.

## Results

### Basic information about the patients

We initially collected bile fluid samples from 26 patients. The demographic data of these patients revealed that 14 samples were obtained from choledocholithiasis patients (6 females, mean age 65 with a range of 31 to 77) and 12 samples were obtained from HCC patients (3 females, mean age 57 with a range of 35 to 74). The levels of ALT, AST, AFP, T-bil, Ferritin, and HBeAg were significantly elevated, while the levels of Albumin and CHE were markedly reduced in HCC patients compared to choledocholithiasis patients. There were no significant differences in sex, age, ALP, γ-GGT, urea, and creatinine between the two groups of patients (Table 1). Furthermore, we collected serum samples from additional 35 patients. The data from these 35 patients indicated that 20 samples were obtained from choledocholithiasis patients (8 females, mean age 60.5 with the range of 31–81), and 15 samples were obtained from HCC patients (6 females, mean age 60 with the range of 43–78). The levels of AFP, Ferritin, and HBeAg were notably increased, while the levels of Albumin and CHE were significantly decreased in HCC patients compared to choledocholithiasis patients. There were no significant changes in sex, age, ALT, AST, T-bil, ALP, γ-GGT, urea, and creatinine between the two groups of patients (Table 2).

**Table 1:** Demographic characteristics, liver function test values, tumor markers, and renal function indicators of patients in the discovery cohort

Variables	Choledocholithiasis (14)	HCC (12)	*p* value
Sex (F/M)	6/8	3/9	NS
Age (year)	65 (31–77)	57 (35–74)	NS
ALT (μ/L)	44.5 (5.5–82.1)	106.5 (37–221.3)	**0.002**
AST (μ/L)	62.5 (11.6–126.6)	118 (62.1–220.4)	**0.003**
Albumin (g/L)	37 (28.47-53.8)	32 (22.7–43.8)	**0.012**
AFP (ng/mL)	0.87 (0.24–2.86)	332 (0.35–1000)	**<0.001**
T-bil (μmol/L)	27.6 (7.7–294.9)	84.5 (15.9–799)	**0.04**
CHE (U/L)	5547 (1033.3–7539.7)	2648.5 (1337.2–7889.9)	**0.024**
Ferritin (ng/ml)	152 (4–300.2)	451.1 (55.2–1650)	**0.013**
ALP (μ/L)	249.3 (61.6–1280.1)	172.5 (91–429.7)	NS
γ-GGT (μ/L)	196 (10.9–1857)	122.1 (35.78–245)	NS
Urea (mmol/L)	3.9 (2.02–15.99)	2.9 (1.43–6.36)	NS
Creatinine (μmmol/L)	54.5 (34.5–201.6)	58.6 (40.9–78.9)	NS
HBeAg (P/N)	0/14	3/9	**0.047**

Note: F, female; M, male; P; positive, N, negative; ALT, alanine transaminase; AST, aspartate transaminase; AFP, alpha-fetoprotein; T-bil total bilirubin; CHE, cholinesterase; ALP, alkaline phosphatase; γ-GGT, gamma glutamyl transpeptidase; HBeAg, hepatitis B e-antigen. NS, not significant. Quantitative data are shown as median values and interquartile rang (in parentheses).

**Table 2:** Demographic characteristics and liver function test values of patients in the validation cohort

Variables	Choledocholithiasis (20)	HCC (15)	*p* value
Sex (F/M)	8/12	6/9	NS
Age (year)	60.5 (31–81)	60 (43–78)	NS
ALT (μ/L)	56.2 (22.3–551)	39.7 (12.2–229)	NS
AST (μ/L)	46 (22.7–314)	62.1 (19–324.1)	NS
Albumin (g/L)	37 (29.9–78)	34.7 (28.5–43.3)	**0.000**
AFP (ng/mL)	1 (0.24–21)	5 (0.2–1000)	**0.000**
T-bil (μmol/L)	24.2 (7.1–136.1)	21.4 (5.1–340.7)	NS
Ferritin (ng/ml)	67.5 (8.9–209.7)	448.6 (99.7–1650)	**0.000**
CHE (U/L)	7872.9 (3875.8–11357.9)	3128 (1632.4–5608.6)	**0.000**
ALP (μ/L)	146.5 (55.5–1242.7)	207.8 (102.1–587.7)	NS
γ-GGT (μ/L)	194.7 (25.6–1478.3)	137.8 (23.1–1573.9)	NS
Urea (mmol/L)	4.6 (2.6–9.2)	4.2 (1.5–12)	NS
Creatinine (μmmol/L)	64.3 (3.5–104.2)	58.6 (3.59–113.7)	NS
HBeAg (P/N)	0/20	7/8	**0.0006**

Note: F, female; M, male; ALT, alanine transaminase; AST, aspartate transaminase; AFP, alpha-fetoprotein; T-bil total bilirubin; CHE, cholinesterase; ALP, alkaline phosphatase; γ-GGT, gamma glutamyl transpeptidase; HBeAg, hepatitis B e-antigen. NS, not significant. Quantitative data are displayed as median values and interquartile rang (in parentheses). As show in Suppl. Fig. 1.

### PRDX2 is a potential diagnostic biomarker of HCC

**Figure 1:**
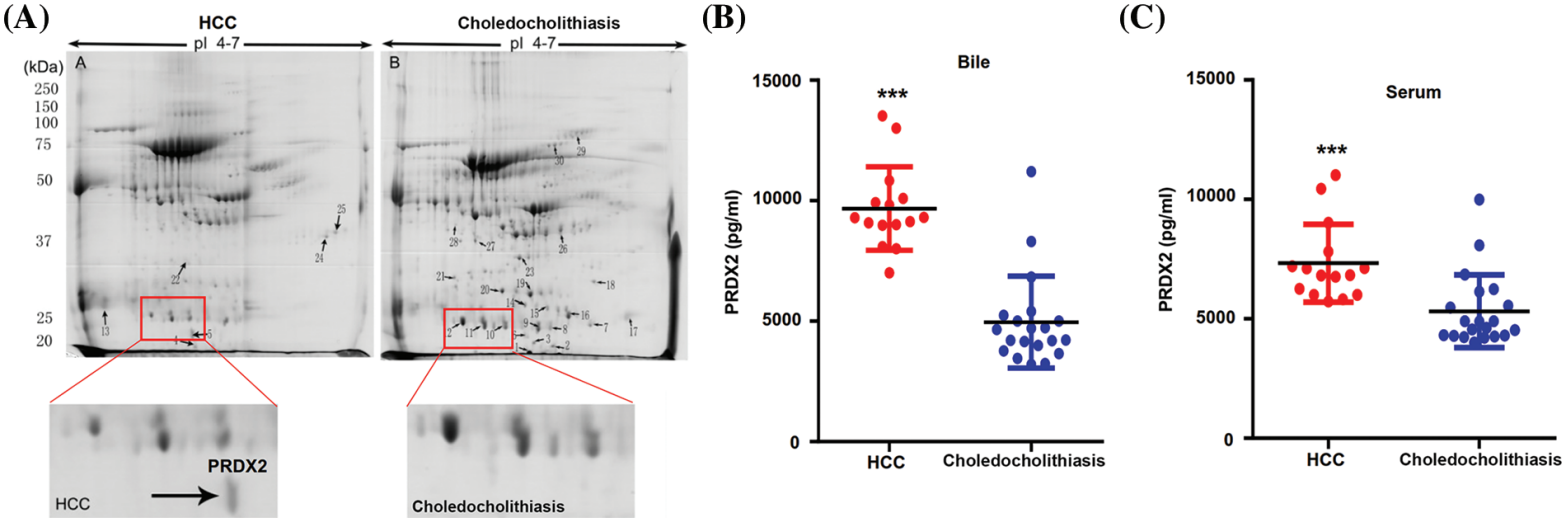
PRDX2 was a potential diagnostic biomarker of HCC. (A) Differential proteomic analysis of bile from HCC (left panel) and choledocholithiasis (right panel) using 2-DE gels. Total protein lysates were separated by isoelectric focusing (IEF) using IPG strips (17 cm, pH4-7) in the first dimension followed by 8% SDS-PAGE in the second dimension. Coomassie-stained gels were analyzed using the PD-Quest 2D analysis software. Totally, 30 differentially expressed spots were identified by MS/MS analysis (upper); 2-DE gel images of PRDX2 in bile from HCC and choledocholithiasis (lower). PRDX2 was upregulated more than 2-fold in bile from HCC when compared with the choledocholithiasis. (B and C) PRDX2 expression was measured by ELISA in the bile (B) and serum (C) of patients with HCC and choledocholithiasis. Horizontal lines indicate the medians. ***p < 0.001.

To investigate the potential diagnostic and therapeutic markers for HCC, we analyzed the related proteins extracted from bile samples of four HCC and four paired choledocholithiasis patients in the “discovery” cohort. The proteins were separated using 2-DE and stained with Coomassie brilliant blue. By employing MALDI-TOF-MS and peptide mass matching, we identified the differentially expressed protein spots in four sample pairs (Fig. 1A). And there was a total of 10 identified differentially expressed proteins. Among them, three proteins (Peroxiredoxin-2, Afamin, and Alpha-1-antitrypsin) were up-regulated in HCC, while seven were down-regulated (Table 3). The molecular mass/pI value of PRDX2 was determined to be 20.209 kDa/8.9, corresponding to spot 5 on the 2-DE gel. The MS/MS spectrum of PRDX2 was shown in Suppl. Fig. 1. Further experiments illustrated a significantly higher PRDX2 level in both bile and serum samples from the full set of the “validation” cohort. In bile, the expression level of PRDX2 in HCC patients was markedly higher than that in choledocholithiasis patients (median level 9,395 *vs*. 4,858 ng/ml, *p* = 0.000) (Fig. 1B). Similarly, the level of PRDX2 in serum of HCC patients was notably higher than that in choledocholithiasis patients (median level 5,780 *vs*. 3,743 ng/ml, *p* = 0.000) (Fig. 1C). Taken together, these data indicated that PRDX2 held potential as a diagnostic biomarker of HCC.

**Table 3:** Proteins identified by mass spectrometry

Accession No.	Gene name	Protein name	Mass (kDa)	pI value	Protein score
IPI00022420	RBP4	Retinol-binding protein 4	23337	5.76	296
IPI00878282	CUBN	Cubilin	23414	5.93	337
IPI00909207	PRDX2	Peroxiredoxin-2	20209	8.90	261
IPI00339263	GGT1	Gamma-glutamyltranspeptidase1	24122	5.26	145
IPI00965913	AFM	Afamin	53076	6.45	371
IPI00964000	IGJ	Immunoglobulin J chain	18509	5.41	179
IPI00815665	PRSS2	Trypsin-2	26927	4.78	248
IPI00745872	ALB	Serum albumin	71317	5.92	488
IPI00869004	SERPINA1	Alpha-1-antitrypsin	34905	5.04	174
IPI00004573	PIGR	Polymeric immunoglobulin receptor	84429	5.58	248

### PRDX2 is a clinic relevant oncogene in HCC

**Figure 2:**
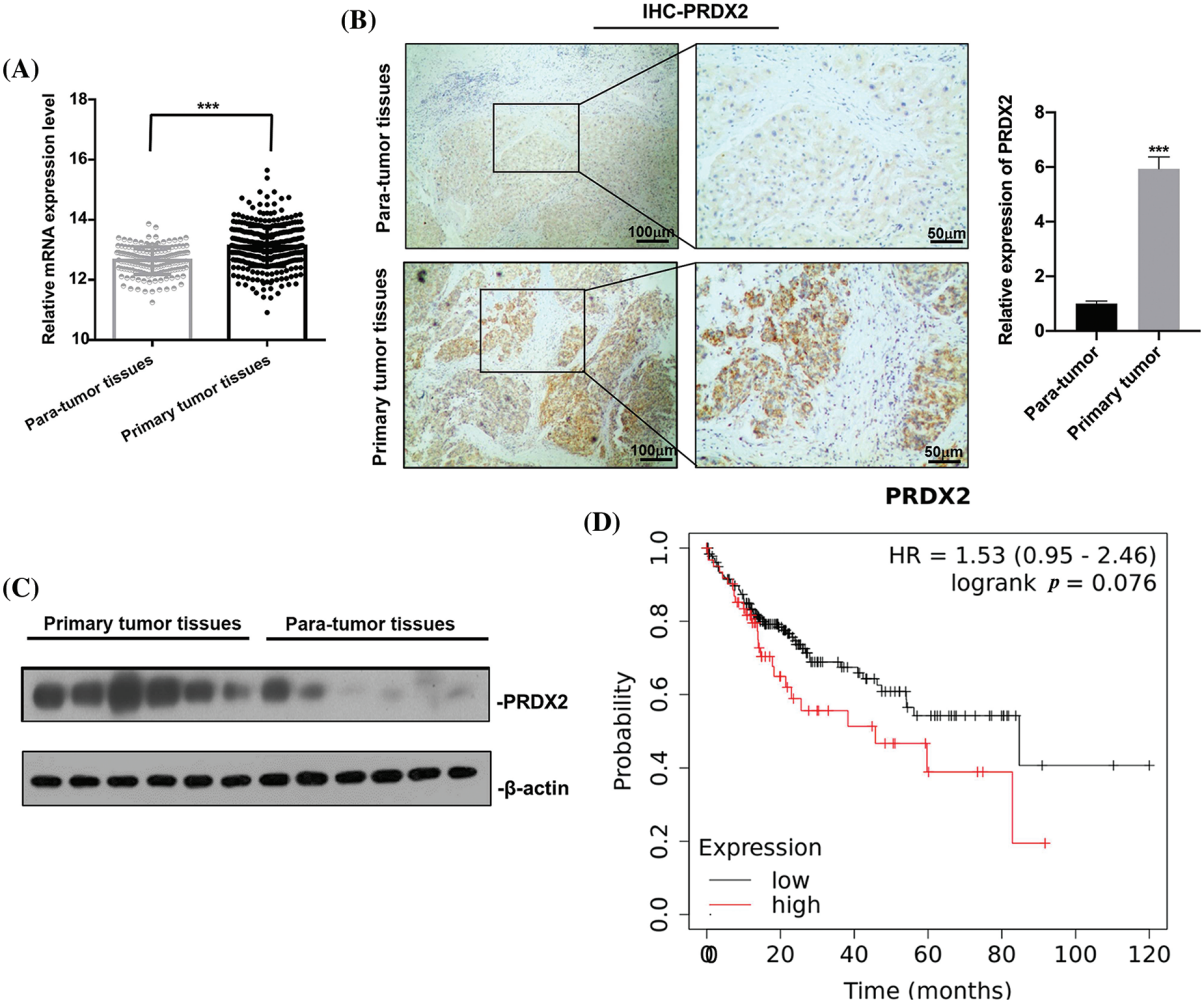
PRDX2 is a clinic relevant oncogene in HCC. (A) PRDX2 mRNA expression in liver cancer tissue and para-tumoral tissue among the HCC patients from TCGA database. (B) IHC results of PRDX2 expression in HCC tissues (primary tumor tissues) and adjacent non-tumor/normal liver tissues (para-tumor tissues), scale bars: 50, 100 μm; magnification: 100× or 400×. (C) Western blotting analysis of PRDX2 expression in primary tumor tissues and para-tumor tissues; 20 μg protein of crude bile from each specimen was loaded onto a homemade 12% SDS-PAGE for separation. Proteins were electroblotted onto a nitrocellulose membrane. Western blot was performed using rabbit monoclonal anti-PRDX2. Lanes 1–6, protein of HCC tissues; lanes 7–12, protein of para-tumor tissues. (D) K-M survival analysis of PRDX2 in HCC patients using TCGA database. ***p < 0.001.

To investigate the potential oncogenic role of PRDX2 in HCC, we analyzed TCGA data and examined the expression change of PRDX2 in the tissues of HCC patients, including tumor tissues and adjacent non-tumor tissues. Results revealed that the expression level of PRDX2 in HCC tumor tissues was significantly higher than that in para-tumor tissues (Fig. 2A). Furthermore, we conducted IHC analysis on HCC tissues and para-tumor tissues obtained from the same HCC patients (n = 9). As shown in Fig. 2B, IHC results demonstrated that the expression of PRDX2 was significantly increased in HCC tumor tissues compared to that in para-tumor tissues. Western blot results also confirmed the increased protein level of PRDX2 in HCC tumor tissues (Fig. 2C). Furthermore, the high expression of PRDX2 was also found to be associated with poor survival of HCC patients (Fig. 2D). To sum up, these results indicated that PRDX2 was a clinically relevant oncogene in HCC.

### PRDX2 promotes the proliferation of HUH-7 cells

**Figure 3:**
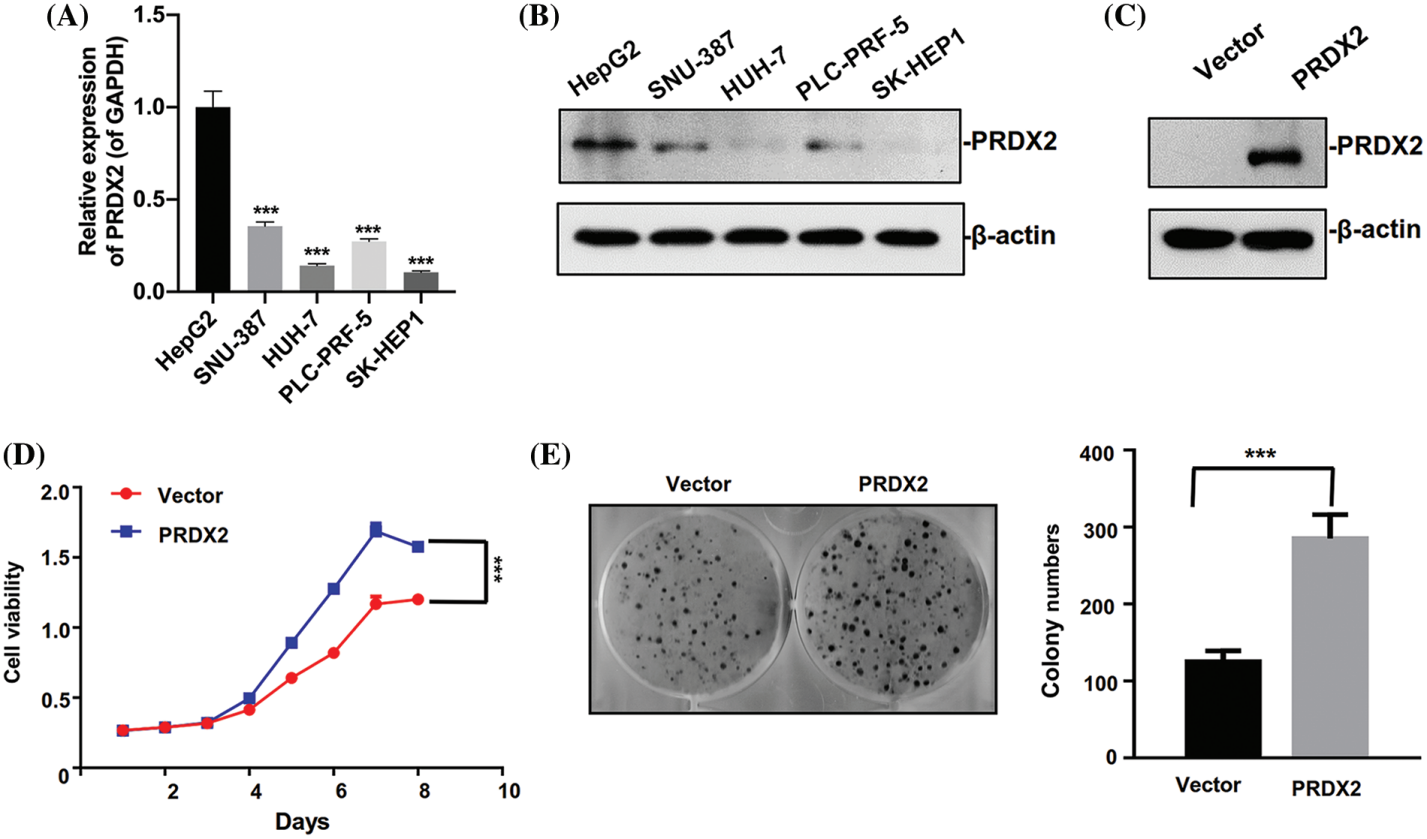
Ectopic expression of PRDX2 promotes HUH7 cells aggressive behavior. (A) RT-qPCR analysis of PRDX2 expression in HepG2, SNU-387, HUH-7, PLC-PRF-5, and SK-HEP1 cells. (B) PRDX2 expression was analyzed using western blot in HepG2, SNU-387, HUH-7, PLC-PRF-5, and SK-HEP1 cell lines. (C) HUH-7 cells were transfected with Vector and PRDX2 for 48 h, and then the cells were treated with puromycin (1 μg/ml) for 48 h, PRDX2 expression was identified with western blot. (D) HUH-7 cells were treated with PRDX2, followed by puromycin (1 μg/ml), and CCK-8 was adopted to assess the effect of PRDX2 overexpression on the viability of HUH-7 cells. OD450 was determined as indicated time points. (E) Effect of PRDX2 overexpression on colony forming ability of HUH-7 cells. After processing with PRDX2 and puromycin, colony forming assay was conducted. ***p < 0.001.

To investigate the oncogene function of PRDX2 *in vitro*, we first analyzed PRDX2 expression in different HCC cell lines. We found that the level of PRDX2 was relatively higher in HepG2 cells than that in other cells including SNU-387, HUH-7, PLC-PRF-5, and SK-HEP1 cells, and the expression level of PRDX2 was lowest in HUH-7 cells (Figs. 3A and 3B). Subsequently, we transfected HUH-7 cells with a PRDX2 overexpression plasmid or a control vector, followed by puromycin selection. Western blotting data confirmed that compared to the vector group, there was a significant increase in PRDX2 expression in the PRDX2-overexpression group (Fig. 3C). To further assess the impact of PRDX2 on the proliferation of HUH-7 cells, we performed CCK-8 and colony forming assays. As shown in Figs. 3D and 3E, overexpression of PRDX2 promoted the growth and colony forming ability of HUH-7 cells.

### Silence of PRDX2 prevents the proliferation of HepG2 cells

**Figure 4:**
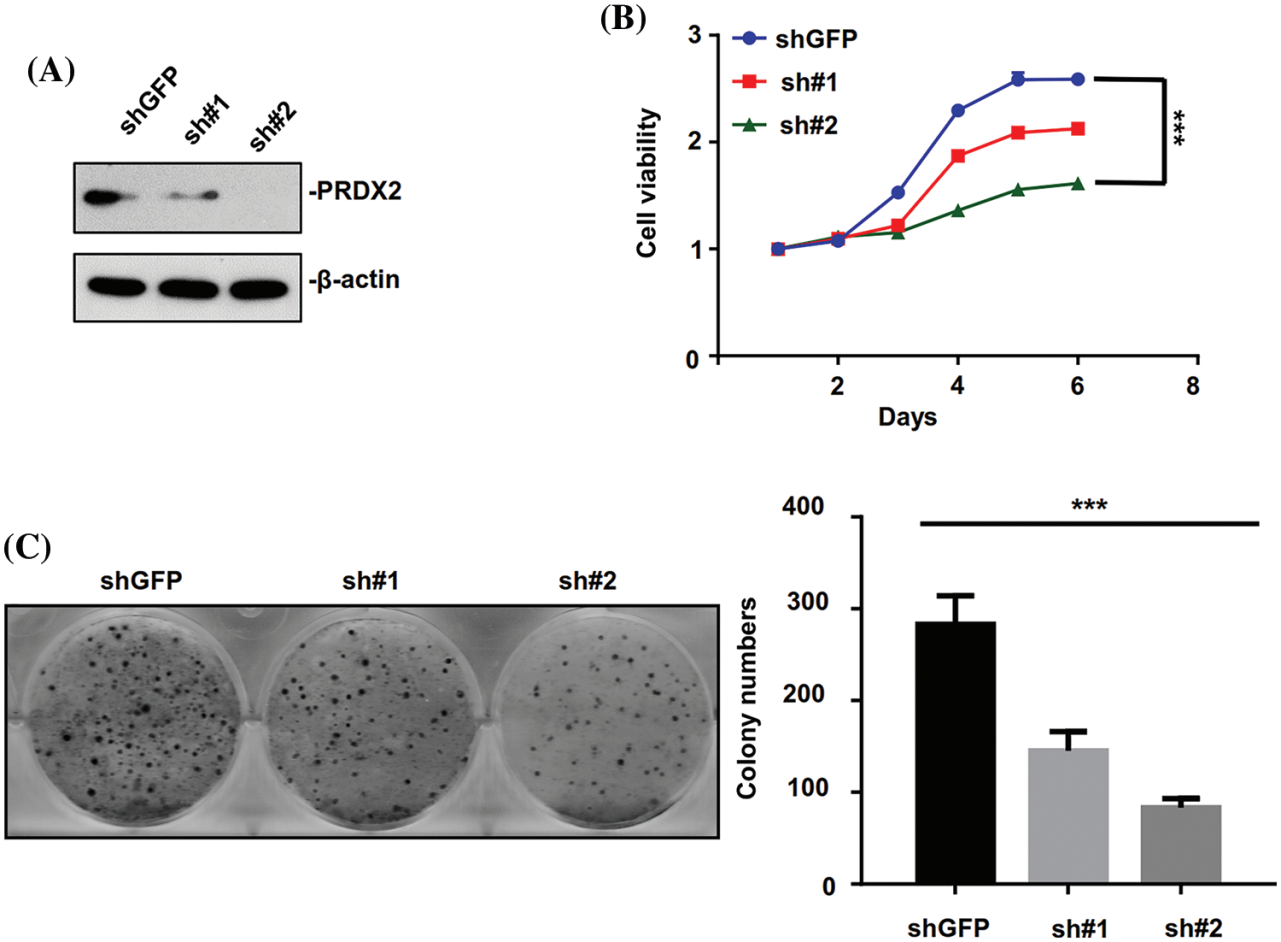
PRDX2 silencing inhibits the aggressive behavior of HepG2 cells. (A) HepG2 cells were transfected with shGFP, pRDX2-shRNA-#1 (sh#1), and PRDX2-shRNA-#2 (sh#2) for 48 h, and then the cells were treated with puromycin (1 μg/ml), PRDX2 expression was monitored with western blot. (B) After processing with sh#1, sh#2, or puromycin, cell viability was confirmed with CCK-8. (C) The proliferation ability was examined using colony forming assay in HepG2 cells after treatment with shGFP, sh#1, sh#2, or puromycin. ***p < 0.001.

Based on the above results, the expression of PRDX2 was found to be highest in HepG2 cells relative to other HCC cell lines. Therefore, the HepG2 cell line was chosen to investigate the impact of PRDX2 knockdown on the relevant function of HCC cells. HepG2 cells were transfected with PRDX2-shRNA-#1 (sh#1) and PRDX2-shRNA-#2 (sh#2). Western blot results indicated that knockdown of PRDX2 efficiently reduced its expression in HepG2 cells, especially with the transfection of sh#2 (Fig. 4A). Subsequently, our results demonstrated that knockdown of PRDX2 led to a decrease in the viability and colony forming ability of HepG2 cells, especially with sh#2 (Figs. 4B and 4C). Taken together, our results showed that PRDX2 contributed to the proliferation of HCC cells.

### Silence of PRDX2 induces senescence and weakens activation of the Wnt/β-catenin pathway in HCC cells

**Figure 5:**
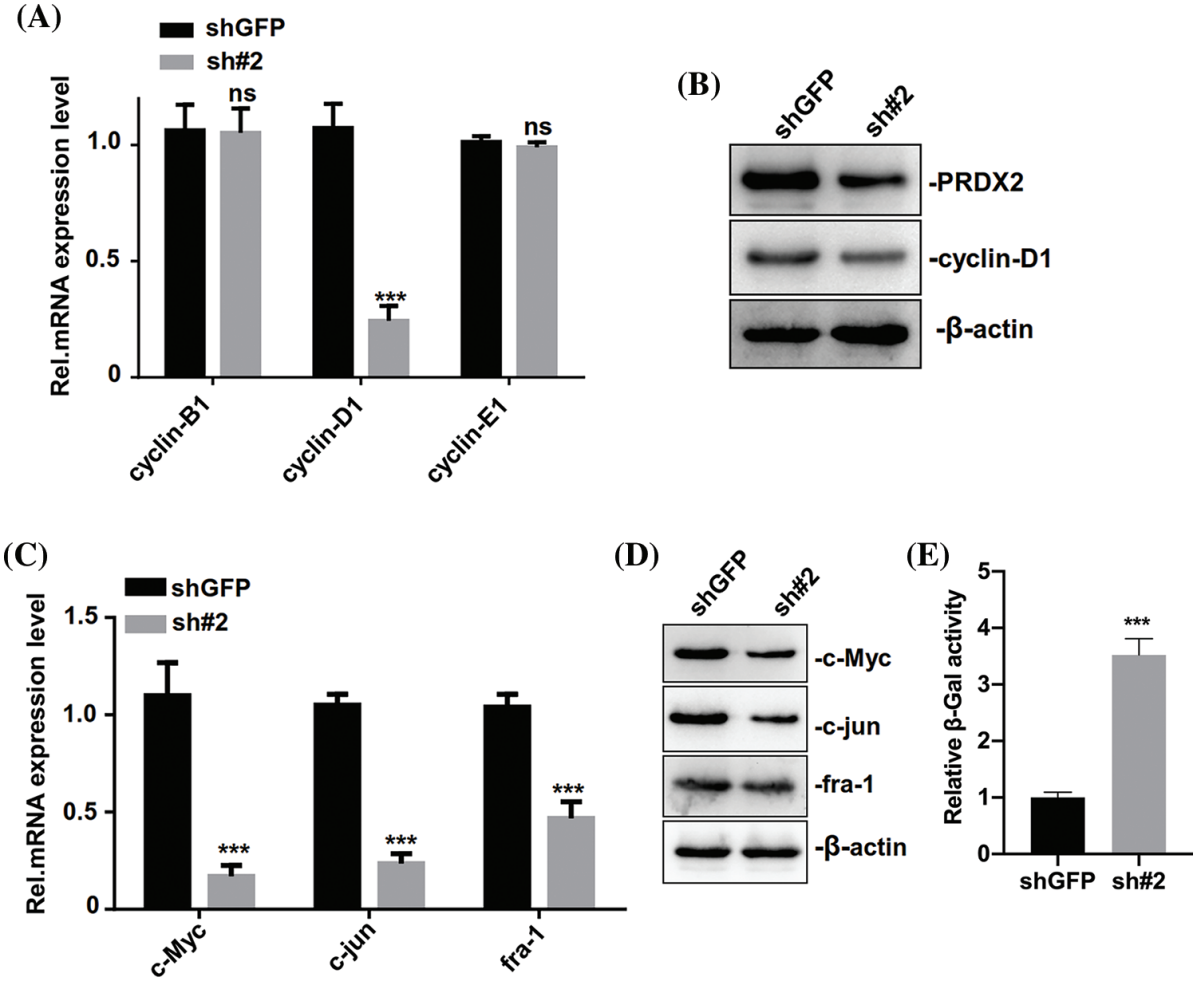
PRDX2 silencing regulates the senescence of HCC cells. (A) HepG2 cells were addressed with shGFP or sh#2 and puromycin, then the levels of cell cycle-related proteins (cyclin-B1, cyclin-D1, and cyclin-E1) were detected by western blot. (B) PRDX2 and cyclin-D1 expressions were determined using western blot in the treated HepG2 cells. The levels of c-Myc, c-jun, and fra-1 were also analyzed with RT-qPCR (C) and western blot (D) in the processed HepG2 cells. (E) The senescence (β-Gal activity) of HepG2 cells was assessed using a β-Gal assay kit. ***p < 0.001.

We further investigated the relevant molecular mechanism of PRDX2 and its impact on the senescence of HCC cells. Through the detection of cycle-related genes (cyclin-B1, cyclin-D1, and cyclin-E1), RT-qPCR data first revealed that the level of cylinD1 was significantly reduced in the PRDX2 silencing group compared to that of the shGFP group (Fig. 5A). And Western blot results also signified that silence of PRDX2 markedly downregulated PRDX2 and cylinD1 in HepG2 cells (Fig. 5B). Since cylinD1 is a downstream transcriptional target gene of the Wnt/β-catenin pathway, we speculated that PRDX2 may be involved in the regulation of this pathway. RT-qPCR and Western blot results further confirmed that knockdown of PRDX2 resulted in a significant downregulation of Wnt/β-catenin pathway-related proteins (c-Myc, c-jun, and fra-1) in HepG2 cells (Figs. 5C and 5D). Furthermore, we found that there was a marked increase in cellular senescence (β-Gal activity) of HepG2 cells after PRDX2 silencing (Fig. 5E). Taken together, these data indicated that PRDX2 silencing could induce the senescence and inhibit the Wnt/β-catenin pathway in HCC cells.

### PRDX2 silencing inhibits HCC tumors growth in vivo

**Figure 6:**
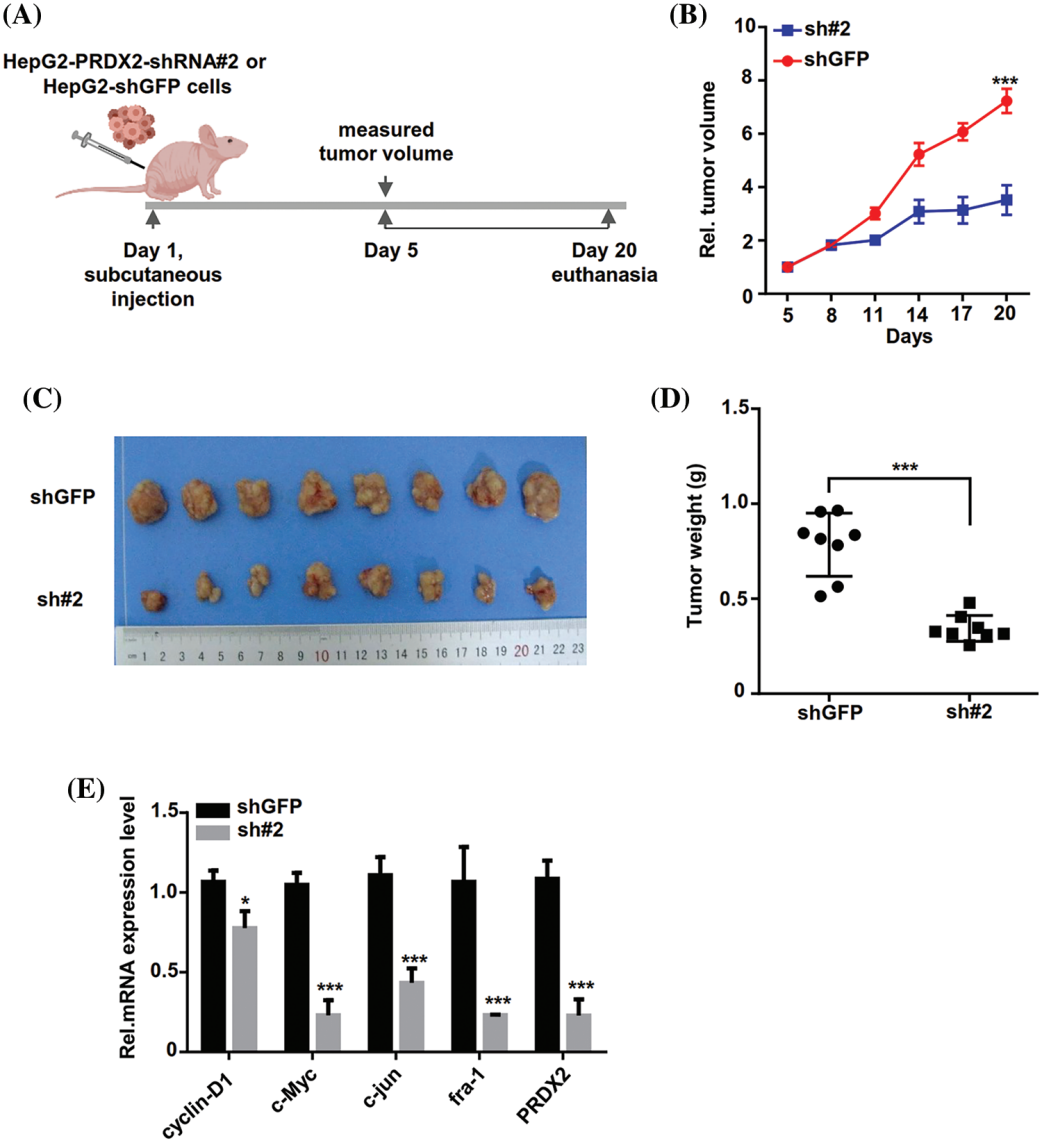
PRDX2 silencing inhibits HCC tumors growth *in vivo*. (A) Schematic illustration of the experimental design of the xenografted tumor in nude mice. (B and C) Effect of sh-PRDX2 on HCC xenografted tumors. Stably HepG2-shGFP (shGFP) and HepG2-sh#2 cell lines were subcutaneously inoculated into nude mice. 5 days week later, when the tumor volume was about 80 mm^3^, the tumor size was measured every three days and the tumor growth curve was drawn. The tumor volume and tumors were displayed. Tumor volume (mm^3^) = d^2^/2 × D (D is the longest, d is the shortest diameter). (D) Statistics of the transplanted tumors weight. (E) The levels of cyclin-D1, c-Myc, c-jun, fra-1, and PRDX2 were monitored using RT-qPCR in the xenografts. *p < 0.05, ***p < 0.001.

To further investigate the *in vivo* function of PRDX2, we inoculated stable HepG2-shGFP (shGFP) and shRNA#2 cell lines into nude mice subcutaneously. Tumor volume was measured every three days and when it reached approximately 80 mm^3^, the mice were euthanized by cervical dislocation 20 days after subcutaneous injection (Fig. 6A). As displayed in Fig. 6B, PRDX2 silencing significantly inhibited tumor growth in mice. Consistently, the tumor size and weight in the shRNA#2 group were significantly smaller than that in the shGFP group (Figs. 6C and 6D). Importantly, the levels of cylinD1, c-Myc, c-jun, fra-1, and PRDX2 were notably down-regulated in the sh#2 group compared to the shGFP group (Fig. 6E). These data strongly argued that PRDX2 silencing inhibited HCC development in a mouse model of HCC.

### PRDX2 promotes β-catenin nuclear translocation

**Figure 7:**
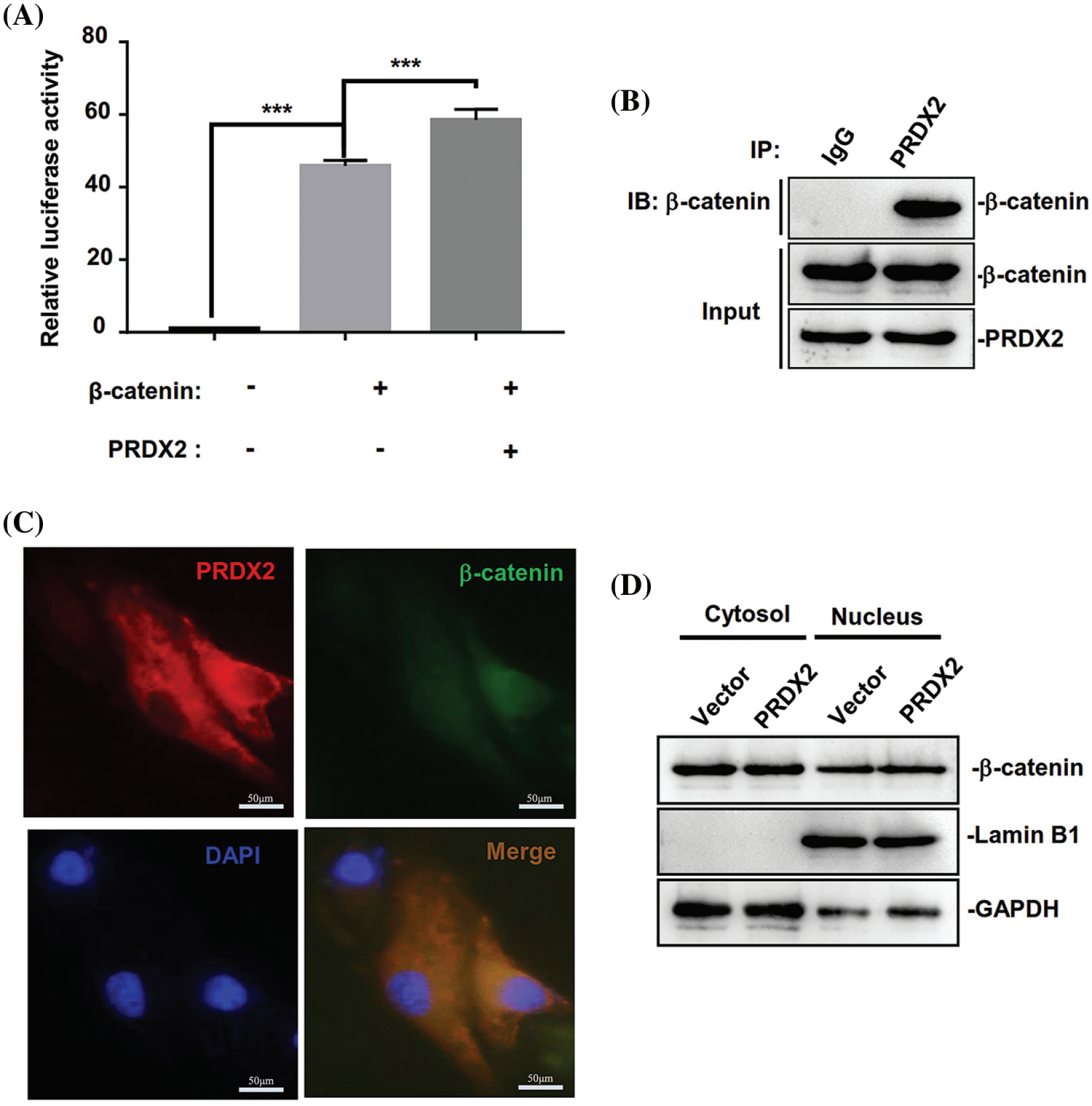
PRDX2 regulates Wnt/β-catenin pathway by promoting β-catenin nuclear translocation. (A) The impact of PRDX2 on β-catenin transcriptional activity. (B) The interaction between PRDX2 and β-catenin was detected by Co-IP experiment. (C) The immunofluorescence results of PRDX2 and β-catenin co-localized in the cytoplasm of HepG2 cells. Red: PRDX2, green: β-catenin, blue: nucleus; scale bars: 50 μm; Magnification: 400×. (D) β-catenin expression in the nucleus and cytoplasm was assessed using western blot after PRDX2 overexpression. ***p < 0.001.

Then we further investigated the underlying molecular mechanism of PRDX2 in regulating the Wnt/β-catenin pathway. We first performed Top-flash assay to assess the effect of PRDX2 on the transcriptional activity of β-catenin. A Top-flash assay results showed a noticeable promotion of β-catenin’s transcriptional activity by PRDX2 (Fig. 7A). Furthermore, Co-IP data manifested the interaction between PRDX2 and β-catenin in HepG2 cells (Fig. 7B). Immunofluorescence results demonstrated the co-localized of PRDX2 and β-catenin in the cytoplasm of HepG2 cells (Fig. 7C). Additionally, the data disclosed that overexpression of PRDX2 markedly increased the expression of β-catenin in the nucleus of HepG2 cells, suggesting that PRDX2 markedly facilitated the translocation of β-catenin into the nucleus (Fig. 7D). Taken together, these data suggested that PRDX2 promoted the activation of the Wnt/β-catenin pathway by inducing the nuclear translocation of β-catenin in HCC cells.

## Discussion

The main reason for the lack of sufficient therapy options for HCC patients is our limited understanding of the molecular mechanism underlying HCC pathogenesis [2]. In our study, we first performed 2-DE and LC-MS on a subset of 26 patients (HCC: *n* = 12, choledocholithiasis (choledocholithiasis): *n* = 14) to identify differentially expressed proteins between HCC and choledocholithiasis patients. We aimed to discover a potential HCC diagnostic biomarker in bile. Our results revealed a significantly higher expression of PRDX2 in the bile of HCC patients compared to choledocholithiasis patients. Similarly, in serum samples, PRDX2 level was notably elevated in HCC causes compared to choledocholithiasis. Furthermore, immunostaining analysis demonstrated distinct differences in staining intensity and the number of positively stained cells between HCC tissues and non-tumor tissues. Therefore, we hypothesized that PRDX2 may function as an oncogene in HCC.

PRDX2 has been identified as a vital cellular defense system against oxidative stress due to its catalytic activity and protein sequence [39]. Accumulating evidence suggests that PRDX2 expression is elevated in a variety of human malignancies [15,16]. Several studies have confirmed the oncogenic role of PRDX2 in different types of cancer. For instance, PRDX2 silencing has been shown to inhibit proliferation and invasion in non-small cell lung cancer cells [14,40], promote growth, survival, and resistance to cisplatin in gastric cancer cells [11,15] and impair proliferation, cell cycle, and autophagy in colorectal cancer cells [11,16]. Additionally, PRDX2 has been implicated in HCC, where its silencing increases ROS production induced by H_2_O_2_, thereby contributing to carcinogenesis [41]. ROS also plays a critical role in the initiation and progression of HCC [42]. Consistent with previous research, our study proved the upregulation of PRDX2 in HCC tissues and its ability to enhance proliferation, supporting its oncogenic function in HCC. Furthermore, considering the involvement of cellular senescence in suppressing HCC cell proliferation, it is plausible that PRDX2 may be implicated in senescence [43]. Our findings align with this hypothesis, as we observed that knockdown of PRDX2 induced senescence in HepG2 cells.

Studies also revealed that PRDX2 possesses the ability to inhibit ROS production in HCC cells by activating Wnt/β-catenin pathway [44]. The Wnt/β-catenin pathway is a classical pathway that regulates the stability and nuclear localization of β-catenin [23]. This pathway plays a key role in cell proliferation, differentiation, and tissue homeostasis maintenance [45]. Aberrations in this pathway can promote cancer stem cell renewal, cell proliferation, and differentiation, thereby significantly contributing to tumorigenesis and treatment response [46]. β-catenin serves as a positive regulator of the Wnt pathway. Upong entering the nucleus, it binds to target genes and initiates the transcription process, regulating the expression of specific genes [47]. The ultimate outcome of Wnt pathway is the binding of β-catenin to T cell transcription factor (Tcf) and subsequent activation of related transcription processes. Studies have proved that introducing exogenous Wnt signaling or mutant genes into normal cells can lead to the formation of β-catenin-Tcf complex, greatly increasing the transcriptional activity of cells. The binding of β-catenin to Tcf may drive the transformation of normal cells into cancerous cells. The accumulation of free β-catenin protein in the cytoplasm and its translocation into the nucleus are key factors in the carcinogenic process associated with aberrant Wnt signaling pathway [48]. Studies also have identified c-Myc as one of the vital oncogenes regulated by aberrant Wnt signaling in the nucleus [49]. Several studies have also revealed the involvement of Wnt/β-catenin pathway activation in the development of HCC [28,50]. In our study, we further revealed that PRDX2 interacted with β-catenin. This interaction stabilized β-catenin and enables its entry into the nucleus of HCC cells, thereby activating Wnt/β-catenin pathway.

In summary, the findings of this study supported the role of PRDX2 as an oncogene in HCC and suggested its involvement in β-catenin nuclear translocation. These results contributed to the theoretical understanding of HCC pathogenesis and provided a foundation for the development of potential therapeutic targets. However, it is important to acknowledge the limitations of this study. The precise mechanism by which PRDX2 affects HCC progression through β-catenin nuclear translocation remains unclear and requires further investigation, which could be addressed through future rescue experiments.

## Supplementary Materials

**Supplementary Figure 1:**
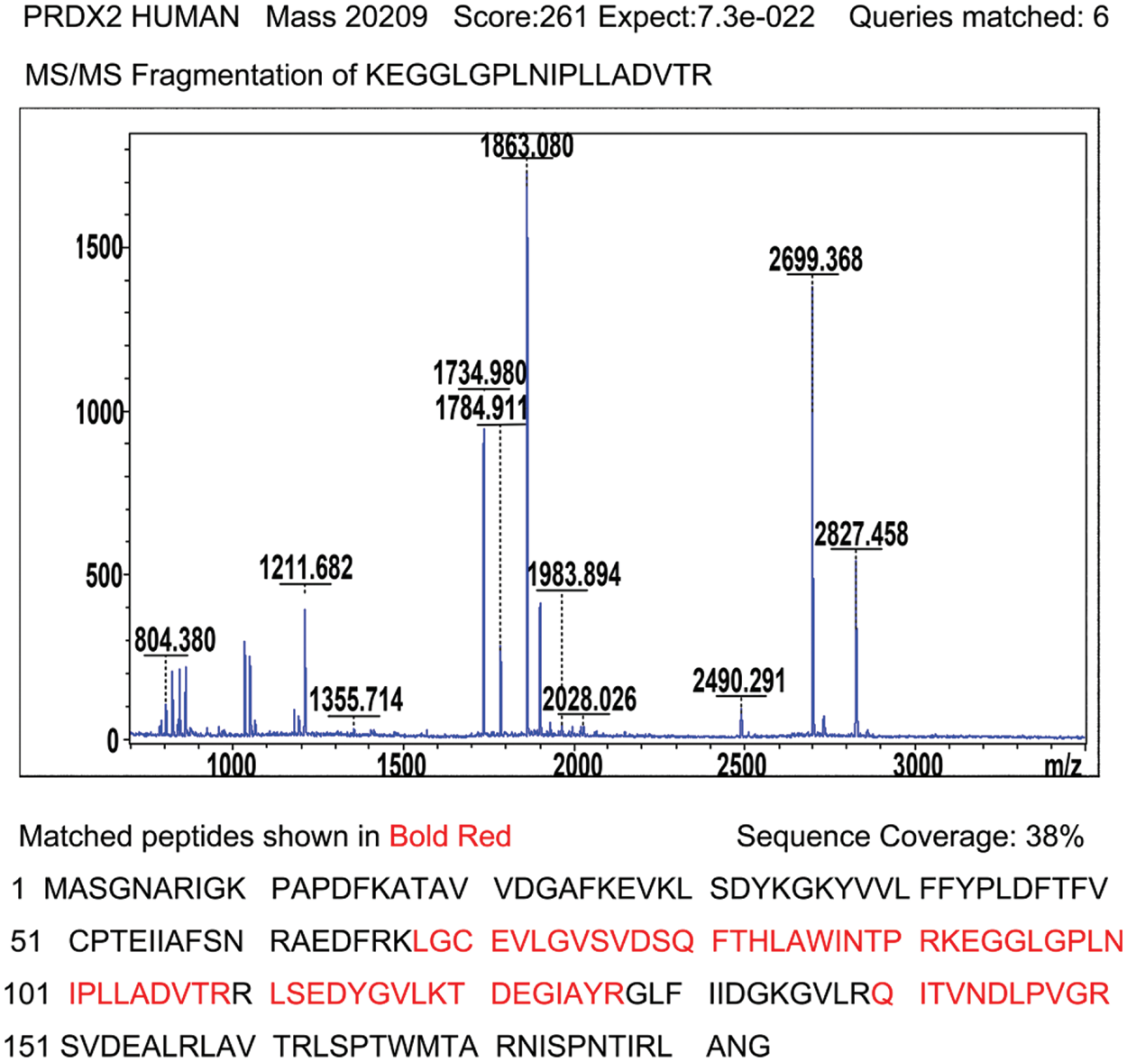
Identification of PRDX2 by MALDI-TOF tandem mass spectrometry. After enzyme digestion, protein identification with high score (261) shows that spot mentioned above corresponds to peroxiredoxin-2; acquired spectra were search against a Mascot Search engine based on the Swiss-Prot protein database; MS/MS spectrum of peptide 92KEGGLGPLNIPLLADVTR109 with corresponding peak values was identified as PRDX2; Schematic representation of the matched peptides in a reference sequence shows 38% of protein coverage.

## Data Availability

The original contributions presented in the study are included in the article/Supplementary Materials. Further inquiries can be directed to the corresponding author.
